# Adult attachment and social anxiety: The mediating role of emotion regulation strategies

**DOI:** 10.1371/journal.pone.0207514

**Published:** 2018-12-28

**Authors:** Darryl L. Read, Gavin I. Clark, Adam J. Rock, William L. Coventry

**Affiliations:** School of Behavioural, Cognitive & Social Sciences, University of New England, Armidale, NSW, Australia; University of Texas Southwestern Medical Center, UNITED STATES

## Abstract

Despite extensive evidence relating attachment dimensions to maladaptive interpersonal behaviours and dysfunctional emotion regulation strategies, few studies have explored social anxiety in the context of adult attachment dimensions. The aim of the present study was to investigate whether attachment-related anxiety and avoidance are associated with symptoms of social anxiety and whether cognitive emotion regulation strategies (reappraisal and suppression) play a role in the relationship between adult attachment and social anxiety. A sample of 253 adults (male n = 47, 18.6%; female n = 202, 79.8%; gender not disclosed n = 4, 1.6%) ranging in age from 18 to 74 years (M = 33.12, SD = 11.56) completed an online questionnaire that consisted of the Experience in Close Relationships–Revised Questionnaire (ECR-R); The Inventory of Interpersonal Situations Discomfort scale (IIS-D); and The Emotion Regulation Questionnaire (ERQ). Results indicated that both attachment anxiety and attachment avoidance have a direct effect on indices of social anxiety symptomology. Reappraisal partially mediated the relationship between attachment anxiety and social anxiety. However, the relationship between attachment avoidance and social anxiety was not mediated by the use of reappraisal and suppression. Findings of the study have implications for the development of clinical interventions targeting mediators of psychological distress associated with social anxiety.

## Introduction

*Social anxiety* refers to anxiety which occurs in relation to social situations and which is most typically conceptualised as arising from the fear of negative interpersonal evaluation [1,2]. Social Anxiety Disorder (SAD) is a common mental illness that is characterized by excessive fear of being scrutinized and judged by others in social situations [3]. For sufferers of SAD, the fear of negative evaluation, potential criticism, or the fear of acting in an embarrassing or humiliating way leads to significant distress and psychosocial impairment. Adults who experience high levels of social anxiety are excessively self-conscious, hold negative core beliefs about themselves, may be unassertive and withdrawn [4] and view themselves as being less socially competent than their peers [5]. Social anxiety has also been demonstrated to be associated with an impaired ability to relate to others [6], and a negative impact on interpersonal functioning, including within romantic relationships [7,8].

Cognitive-behavioural models of social anxiety highlight dysfunctional thought processes about oneself and one’s social world, irrational underlying beliefs and assumptions, interpretive biases, and the use of maladaptive emotion regulation strategies as core features of the disorder (e.g., [9,4]).

Several investigators have suggested that individual *attachment* style and social anxiety disorder are strongly intertwined and that dysfunctional attachment styles may predispose individuals to social anxiety symptomology (e.g., [10–13]). Therefore, adult attachment style may be an important individual difference variable that can influence the onset and maintenance processes of social anxiety.

### Attachment theory

Attachment theory [14–17] is one of the most widely used conceptual frameworks in the study of individual differences in personality and social development. A large body of research has evaluated how individual differences in attachment influence emotion regulation and patterns of interpersonal behaviour within adult relationships (e.g., [18]).

Attachment theory postulates that early experiences give rise to cognitive schemas of the self and others that are embedded with the attachment system. Bowlby [15] refers to these cognitive schemas as internal working models, which are based upon expectations and beliefs about the availability, dependability and supportiveness of attachment figures (i.e., models of others) and whether the self is worthy of attention, care and support (i.e., models of self). These internal working models serve as a template for the development of later interpersonal relationships and are thought to influence the way in which an individual experiences, processes and expresses emotions in all domains of life [19].

If attachment figures are seen as consistently available and responsive to signals of distress, the individual forms a secure attachment whereby they internalize perceptions of others as responsive, caring and trustworthy, and the self as valued and competent (i.e., a positive model of others and the self). By achieving desired outcomes in the attachment relationship, securely attached individuals build confidence in their use of support seeking as a distress regulation strategy, which motivates them to seek proximity to significant others in times of need. Thus, a secure attachment fosters the formation and acquisition of social competencies that are required to establish and maintain supportive and trusting relationships in adulthood [20]. Conversely, if attachment figures are unresponsive, rejecting, and offer inconsistent behavioural responses to distress, the individual develops an insecure attachment whereby they internalize perceptions of others as unreliable and untrustworthy (i.e., a negative model of others) or the self as unworthy and ineffective (i.e., a negative model of the self). Insecure attachment fosters the development of insecurities regarding the effectiveness of the use of support seeking as a distress regulation strategy, which interferes with the acquisition of critical social competencies [21].

Although previous attachment research has conceptualised distinct types of attachment orientation in adults (e.g., secure, preoccupied, dismissing, fearful; [22]), a number of authors have argued that adult attachment should be measured dimensionally rather than typologically because it is “a variable on which people vary in degree rather than in kind” [23], (p108). Contemporary models of adult attachment, therefore, conceptualise individual differences as variations along two orthogonal dimensions; that is, *attachment anxiety* and *attachment avoidance* [24]. In this two-dimensional space, individuals displaying an adaptive, or “secure” attachment style, reflect those who demonstrate low levels of both attachment anxiety and attachment avoidance [25].

Individuals with elevated attachment anxiety and/or attachment avoidance are considered to be predisposed to employing dysfunctional emotion regulation strategies [26]. By experiencing repeated failure in their attempts to alleviate distress, insecurely attached individuals develop alternative strategies of distress regulation whereby they elevate their levels of distress in an effort to fulfil their attachment needs (i.e., hyper-activating strategies associated with attachment anxiety) or they deactivate their attachment system by reducing their reliance on the attachment figure in an effort to avoid negative emotional experiences (i.e., deactivating attachment strategies associated with attachment avoidance; [27,28].

Attachment anxiety refers to negative models of the self and reflects the degree to which an individual attempts to minimize distance from others due to fear of rejection or worries regarding the availability and responsiveness of others. The hyper-activating strategies associated with anxious attachment are characterized by energetic attempts to elicit support from others through the use of coercive, clinging and controlling behaviours [25]. Attachment avoidance refers to negative models of others and reflects the degree to which an individual avoids being dependent on others and views others as untrustworthy [24]. The deactivating strategies associated with an avoidant attachment are characterised by the suppression of thoughts and memories that evoke feelings of vulnerability and distress, social withdrawal, interpersonal hostility, and a desire to maintain independence [26,28,29]. Thus, attachment anxiety and avoidance are believed to be differentially associated with maladaptive strategies of emotion regulation.

### Adult attachment and social anxiety

A variety of research has demonstrated that attachment anxiety and attachment avoidance are associated with symptoms of anxiety [30–33]. However, despite the clear conceptual relevance of attachment anxiety to the perception of interpersonal threat, relatively little research has directly investigated the relationship between social anxiety symptomology and adult attachment. The small body of research which has explored this relationship has typically only made distinctions between the securely and insecurely attached adults (e.g., [34]).

To date, no published research has specifically investigated social anxiety symptoms and the dimensions of attachment anxiety and attachment avoidance. The limited research which has been conducted (i.e., research which has investigated attachment security as a categorical variable and which has not evaluated variance on the dimensions of attachment anxiety or avoidance) suggests insecure attachment is associated with stronger symptom severity in social anxiety [10,35,36]. However, it is unclear in what way attachment anxiety and attachment avoidance are associated with symptoms of social anxiety. As noted above, theoretical conceptualisations of attachment suggest that each attachment dimension may impact on level of psychological distress (e.g. depression) but are purported to do so through differential behavioural and affect-regulation mechanisms. As such it would be expected that each attachment dimension would differentially influence social anxiety. However, a wide body of research has demonstrated that variables *relevant* to social anxiety are associated with each of the attachment dimensions and, as such, it may be hypothesised that both attachment domains are related to social anxiety symptoms. For example, adults with high levels of attachment anxiety have been found to report exaggerated threat appraisals in response to stressful life events [26] in daily social interactions [37] and in group interactions [38]. Individuals high on attachment anxiety are conceptualised as reflecting an internalised negative view of one’s worthiness of receiving support, doubt their capacity to cope with distress and present with fears regarding negative interpersonal outcomes (e.g., [25]). As such, they would be expected to display a predisposition to anxiety in social contexts and the fear of negative evaluation. In contrast, research suggests that individuals high in attachment avoidance are highly self-critical [39], exhibit an intolerance of uncertainty [40], view others as untrustworthy (i.e., negative model of others) and are uncomfortable with closeness [41]. Each of these attachment-driven views of self and others would be hypothesised to predispose individuals high in attachment avoidance to experience social interactions as threatening (due to a general tendency to view others’ intentions as negative/untrustworthy) and anxiety provoking (e.g., individuals with intolerance of uncertainty may experience distress surrounding social interactions, which are inherently ambiguous and contain uncertain outcomes; [42]. As such, individuals high on attachment avoidance would also be hypothesized to be vulnerable to social anxiety symptomology and be associated with such individuals’ sensitivity to rejection, though this may be through a distinct process from that which suggests individuals high on attachment anxiety may be vulnerable to social anxiety symptomology. Due to the lack of data examining the relationship between attachment, emotion regulation and social anxiety symptoms, the emotion regulatory processes through which heightened attachment anxiety and/or attachment avoidance might impact upon symptoms of social anxiety are unclear.

### Adult attachment and emotion regulation

As indicated above, heightened attachment anxiety and/or attachment avoidance is believed to predispose individuals to employ maladaptive emotion regulation strategies, which perpetuate attachment-related distress [28]. In support of this contention, adults with high levels of attachment anxiety have been found to be more likely to express their emotions, experience more intense emotions, and regulate feelings of distress within interpersonal relationships through the use of emotion focused coping (i.e., hyper-activating) than those low on attachment anxiety (e.g., [43–45]). Such strategies ultimately increase their distress. Furthermore, adults with high levels of attachment anxiety have been reported to experience difficulties in suppressing unwanted thoughts such as negative social feedback [46], and ruminate on intrusive negative thoughts [47]. In contrast, adults with high levels of attachment avoidance inhibit emotional displays, deny emotional distress, and cope with distress by distancing themselves from the source of distress (i.e., deactivating strategies) [27,48].

Research in the past decade has highlighted the importance of two cognitive emotion regulation strategies; *reappraisal* and *suppression* (e.g. [49]). Reappraisal is considered to be an adaptive emotion regulation strategy and refers to ‘‘reframing a negative emotional event such that the new understanding renders the event less aversive” [50], (p269). This strategy is, therefore, conceptualised as being an antecedent-focussed regulation strategy in that it involves attempts to attenuate distress through altering the impact of emotion generating cues [51]. Reappraisal has been demonstrated to be negatively associated with levels of anxiety symptomology [28,25]. In contrast, suppression, (also commonly referred to as *expressive suppression*), is considered a dysfunctional emotion regulation strategy which involves the inhibition of an already activated emotional response (i.e., response-focused) by down-regulating the outward expression of the emotion [51]. Suppression has been demonstrated to be positively associated with symptoms of anxiety [52–54]. Despite multiple studies showing a link between insecure attachment dimensions and difficulties in emotion regulation [55], there are few studies that have specifically investigated the relationship between anxious and avoidant attachment and the use of suppression and cognitive reappraisal. Conceptually, individuals with secure attachment would be expected to utilise cognitive reappraisal more extensively, and utilise suppression less, than individuals high on attachment anxiety and avoidance. The existing evidence base provides inconsistent support for this contention, with a number of studies suggesting such associations may exist but that different insecure attachment dimensions are associated with different, and distinct, attachment strategies for regulating distress (e.g., [28,56–60]). It has been proposed that attachment avoidance is associated with preferential utilisation of suppression while, in contrast, heightened attachment anxiety is associated with the (ineffective) use of reappraisal [61,62]. Research suggests that individuals high on attachment anxiety experience difficulty withdrawing attentional resources from threatening stimuli which is believed to exacerbate emotional reactivity, leading to deficits in the ability to utilise cognitive reappraisal [22,61].

Attachment avoidance has been found to be more strongly associated with the use of suppression when compared to anxiously attached individuals (e.g., [57,61]). Radecki-Bush et al. [63] found that avoidant individuals rated themselves as having more control when confronted with socially threatening situations when compared to anxious individuals and were, therefore, more likely to use reappraisal as an emotion regulation strategy. Vrticka et al. [62] reported results of an fMRI study in which individuals high on avoidant attachment demonstrated a preferential use of emotion suppression and were not able to effectively engage in cognitive reappraisal when instructed. In contrast, individuals high on attachment anxiety were able to utilise cognitive reappraisal and suppression when instructed to do so.

Results of research investigating the relationship between emotion regulation and social anxiety symptomology may be seen to be more consistent. A small body of experimental research indicates that socially anxious individuals exhibit deficits in their ability to use cognitive reappraisal when confronted with situations that are interpreted as socially threatening [64,65]. There is also evidence that suppression is used excessively and unhelpfully by individuals with heightened social anxiety with research indicating that individuals high in social anxiety report frequently suppressing both positive [62,66,67] and negative emotions [68,69]. However, it is unclear whether factors such as attachment style may influence individual differences in the use of emotion regulation strategies and to what degree the different strategies may be associated with social anxiety symptoms.

### The relationship between attachment, emotion regulation and social anxiety

Fraley et al. [70] argue that variations in the regulation of attachment related goals (i.e., proximity maintenance associated with anxious attachment and avoiding rejection associated with avoidant attachment) account for individual differences in anxiety symptoms and interpersonal distress. Consistent with this notion, past research has shown that higher levels of attachment anxiety and attachment avoidance may predispose individuals to experience a greater degree of negative affect in interpersonal situations [71,72]. Thus, heightened attachment anxiety and/or attachment avoidance would be expected to lead to higher levels of social anxiety symptomology. However, there is reason to hypothesise that this relationship is influenced by the individual use of the emotion regulation strategies of suppression and reappraisal. As noted above, individuals with heightened attachment anxiety and attachment avoidance would be expected to display greater use of dysfunctional emotional regulation strategies (i.e., greater use of suppression and lesser use of reappraisal) relative to individuals low on these dimensions. A growing body of research has demonstrated individuals who exhibit high social anxiety symptoms report less frequent and effective use of adaptive regulation strategies, such as reappraisal, and rely more on maladaptive strategies, such as suppression (e.g., [49,64]). Collectively, this research may suggest that individuals with heightened attachment anxiety and attachment avoidance may be predisposed to employ dysfunctional emotion regulation strategies (i.e., higher suppression and lower reappraisal), which, in turn, will lead to heightened levels of social anxiety symptomology. In order to explore this hypothesis, two mediation models were developed and tested following the precedent established in previous attachment-related research (e.g., [33]). These models proposed two hypothetical causal chains whereby (1) the effects of attachment anxiety on social anxiety symptoms would be mediated by suppression and reappraisal, and (2) the effects of attachment avoidance on social anxiety symptoms would be mediated by suppression and reappraisal. To our knowledge, the present study is the first to evaluate the mediating effects of emotion regulation, specifically cognitive reappraisal and suppression, on the relationships between attachment and social anxiety. Establishing the impact of attachment dimensions on use of emotion regulation and social anxiety symptoms may have important implications for the manner in which social anxiety difficulties are assessed and treated in clinical practice. Obtaining a clearer understanding of these relationships may help to increase the efficacy and specificity of psychological interventions for social anxiety through targeting pertinent negative internal working models and associated maladaptive distress regulation strategies.

### The present study

The present study aimed to evaluate whether the dimensions of attachment anxiety and attachment avoidance are associated with social anxiety symptoms and whether maladaptive emotion regulation strategies (i.e., lower reappraisal and higher suppression) mediate this relationship. Due to the limited research on social anxiety and attachment, the present study aimed to measure general social anxiety symptomology as well as social anxiety symptoms across specific interpersonal situations, as measured by the Inventory of Interpersonal Situations (IIS; [73]). The IIS evaluates overall social anxiety as well as the following five situational subscales: (a) Giving Criticism; (b) Expressing an Opinion; (c) Giving a Compliment; (d) Initiating Contact; and (e) Positive Self-Evaluation.

The following hypotheses were formulated:

1. Attachment avoidance and attachment anxiety would be positively associated with social anxiety, with attachment anxiety displaying a significantly greater correlation;
2. Attachment avoidance and attachment anxiety would be positively associated with the use of suppression, with attachment avoidance displaying a significantly greater correlation;
3. Attachment avoidance and attachment anxiety would be negatively associated with the use of cognitive reappraisal, with attachment anxiety displaying a significantly greater correlation;
4. The relationship between attachment anxiety and social anxiety will be mediated by cognitive reappraisal and suppression; and
5. The relationship between attachment avoidance and social anxiety will be mediated by cognitive reappraisal and suppression.

## Method

### Participants

A total of 296 adults aged 18 years and older participated in the study. Forty- three (14.52%) participants were excluded from the study as a result of significant missing data, yielding a final sample of 253 participants. This sample comprised 47 males (18.6%) and 202 females (79.8%), with four participants (1.6%) choosing not to report their gender. Participants ranged in age from 18 to 74 years (*M* = 33.12, *SD* = 11.56).

### Measures

#### Demographic questionnaire

Participants were asked to report age, gender and level of education.

#### The experience in close relationships–revised questionnaire (ECR-R; [70])

The ECR-R is a 36-item measure of adult attachment that is comprised of two 18-item subscales assessing attachment anxiety and attachment avoidance. Respondents endorse statements on a seven-point Likert scale, (1 = Strongly Disagree to 7 = Strongly Agree) reflecting their feelings regarding close relationships, with higher scores indicating higher attachment anxiety or attachment avoidance. Examples of avoidant and anxious items, respectively, are, “I get uncomfortable when a romantic partner wants to be very close” and “When my partner is out of sight, I worry that he or she might become interested in someone else”. The ECR-R has demonstrated excellent test-retest reliability [74] and shown excellent internal consistency [70]. In the present study, Cronbach’s alpha was .94 for the anxiety subscale and .95 for the avoidance subscale.

#### Inventory of interpersonal situations–discomfort (IIS-D; [73])

The IIS was developed to measure both discomfort in social situations and frequency of occurrence. For the purposes of the present study, only the discomfort scale was used, as this reflects level of social anxiety, and is labelled as social anxiety henceforth within this paper. The IIS-Discomfort (IIS-D) is a 35-item self-report scale which asks respondents to identify the degree of *discomfort* they would experience in 35 different types of social situations on a five-point Likert scale with responses ranging from 1 (none/never) to 5 (very much/always). Examples items include “Joining a conversation of a small group of people” or “Expressing an opinion that differs from that of the person with whom you are talking”. The IIS-D is comprised of the following five subscales: Giving Criticism; Expressing Opinions; Giving Compliments; Initiating Contact; and Positive Self-Evaluation. The overall scale and subscales for Discomfort has been shown to have good validity and reliability in both adult and non-psychiatric samples [73].

Van Dam Baggen et al.[73] report the IIS-D to exhibit excellent internal consistency and good test-retest reliability (*r =* .84). In the present study, the Cronbach’s alpha was .96. Cronbach’s alpha for the subscales were, as follows: Giving Criticism (α = .91); Expressing Opinions (α = .90); Giving Compliments (α = .72); Initiating Contact (α = .83); and Positive Self-Evaluation (α = .79).

**The Emotion Regulation Questionnaire (ERQ;** [51]**)** is a 10-item self-report questionnaire designed to measure respondents’ tendency to regulate their emotions in two ways: (1) Cognitive Reappraisal (six items), and (2) Suppression (four items), with subscales scored as the mean of the items. Responses are scored on a seven-point Likert scale from 1 (“strongly disagree”) to 7 (“strongly agree”). Gross et al. [51] found test-retest reliability of .69 for both the reappraisal and suppression subscales, and internal consistency of each subscale was acceptable (reappraisal, α = .79; suppression, α = .73). In the present study, the Cronbach’s alpha was .84 for reappraisal and .81 for suppression.

### Procedure

The present study was conducted following approval of the Human Research Ethics Committee of the University of New England, New South Wales, Australia.

Participants were recruited by posting the study advertisement, containing a link to the online study, on social media (e.g. Facebook groups) and on the University of New England’s online learning platform. Furthermore, undergraduate Psychology students at the University of New England were given the opportunity to participate in exchange for course credit. After following the link on the study invitation, participants were presented with the study information sheet followed by the survey. The survey was completed anonymously utilizing the Qualtrics software platform (Qualtrics, Provo, UT) and consisted of a demographic questionnaire and the measures described above.

## Results

### Descriptive statistics

Means and standard deviations of the IIS Discomfort scale, when summing the 35 items to compute a total score, were 86.58 and 25.20, respectively, with a minimum score of 35 and a maximum score of 155 (Range = 120). (In the subsequent analyses, we used the mean of the ISS-D rather than the total to align with the other scales, where we also used the mean). The ISS scales have been shown to discriminate between socially anxious and non-socially anxious samples, and are correlated with independent measures of social anxiety [75]. Mean and standard deviations of the overall Discomfort scale have previously been established for socially anxious psychiatric patients (*M* = 100.00, *SD* = 26.10), heterogeneous psychiatric patients (*M* = 91.80, *SD* = 27.80), non-clinical respondents (*M* = 70.50, *SD* = 17.80), and undergraduate students (*M* = 70.90, *SD* = 16.40; [75]). Forty percent of the sample in the present study scored above the reported IIS mean of heterogeneous psychiatric patients and 30% of the sample scored equal to or greater than the reported means for socially anxious patients [75]. Consequently, it can be claimed that the present study’s sample displayed a range of social anxiety symptomology, including a significant proportion displaying social anxiety symptoms at the degree of severity reported within socially phobic samples.

### Analyses

**Fig 1:**
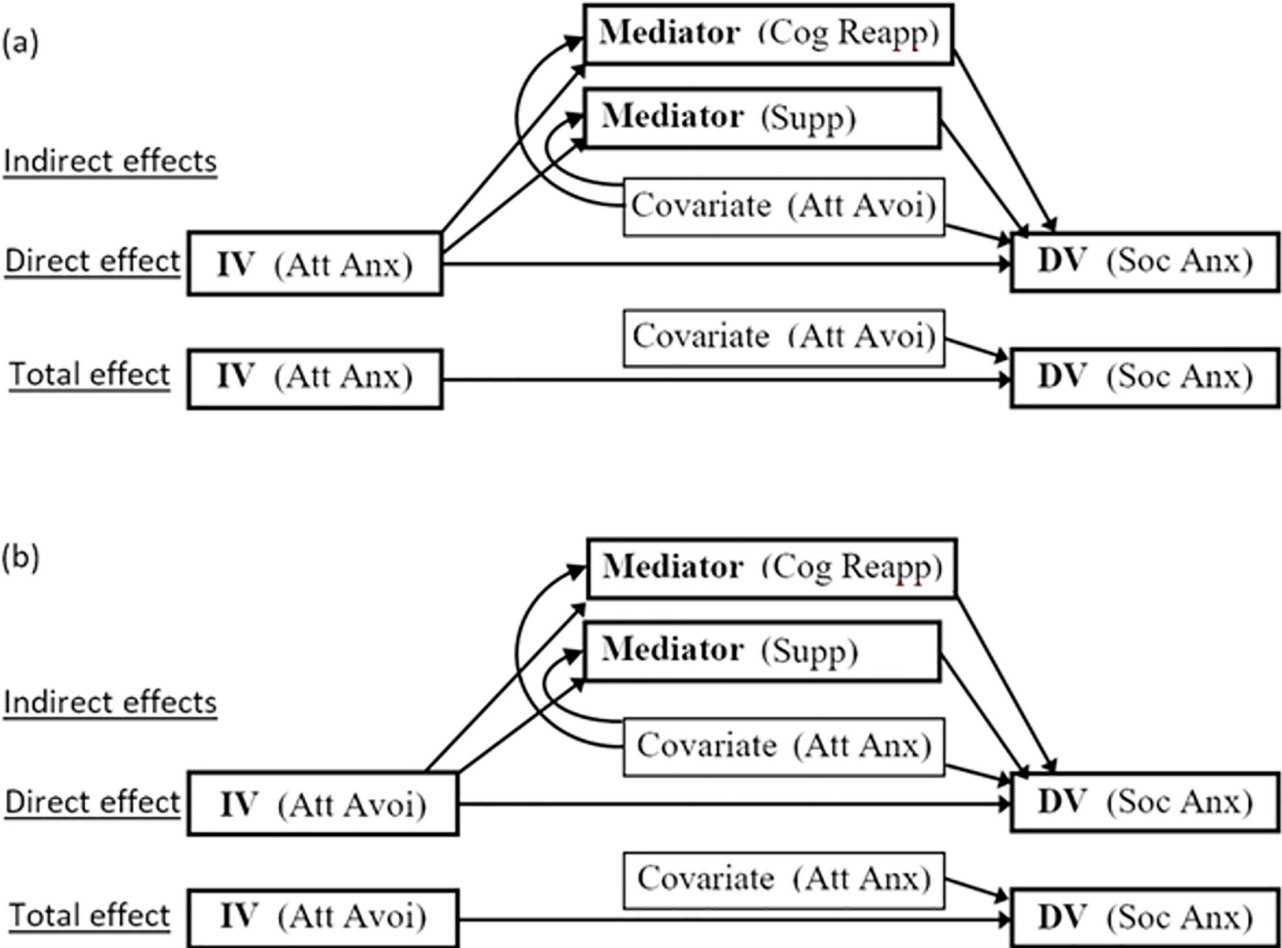
Path diagram mediation models. In the initial mediations (a and b) there was no covariate. Follow-up mediations (a and b) included a covariate, and in those models the paths for both the total effect (IV-DV) and IV-Mediator are interpreted with the effects of the covariate removed, the direct effect (IV-DV) with the covariate and Mediator-DV path removed, and the Mediator-DV path with the covariate and IV-DV path removed. IV = independent variable, DV = dependent variable, Att Anx = attachment anxiety, Att Avoi = attachment avoidance, Cog Reapp = cognitive reappraisal, Supp = suppression, Soc Anx = social anxiety.

**Fig 2:**
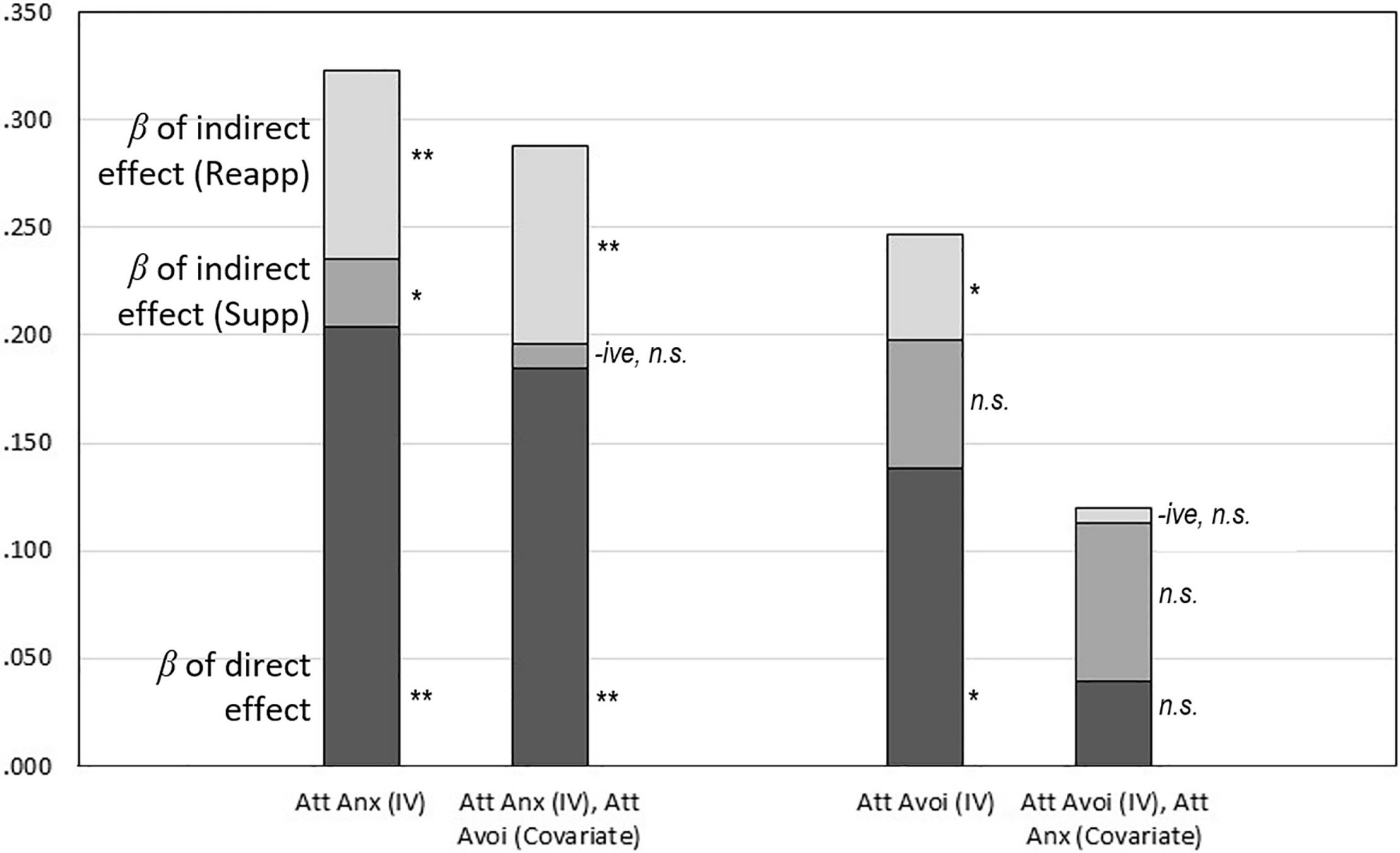
The relative sizes of the direct and indirect effects as indicated by the standardized beta weights (*β*) when presented as absolute values. Two betas were negative (*-ive*), as noted to the left of the bars. The figure shows four mediations, all had social anxiety as the dependent variable and two mediators, cognitive reappraisal (Reapp) and suppression (Supp). The independent variable (IV) was either attachment anxiety (Att Anx) or attachment avoidance (Att Avoi), as indicated, and the covariates, when included, were either attachment anxiety or attachment avoidance. Significance levels: * *p* < .05, ** *p* < .01, *n*.*s*. = not significant.

All hypotheses were assessed against an alpha level of .05. Hypotheses 1 to 3 were evaluated with Bivariate Pearson correlations and multiple regressions, with differences in correlation coefficients evaluated through converting *r* values to *z* scores following the process outlined by Lee et al. [76]. Hypotheses 4 and 5 were evaluated using two mediations. Each mediation comprised of three analyses: (1) testing the indirect effect with a mediation performed using the PROCESS add-on in SPSS, selecting model 4 and stipulating the independent variable (IV), mediator and dependent variable (DV) (detailed in Fig 1); (2) testing the total effect with a regression run in SPSS between the IV and DV; and (3) testing the direct effect with a regression in SPSS using two variables, the IV and mediator, to predict the DV. Ordinarily, it is only necessary to run the first analysis (i.e., the mediation in PROCESS). However, the strategy above was adopted to compare the standardized beta weights (*β*s) of the total, direct and indirect effects (illustrated in Fig 2) and PROCESS only produces *β* for the indirect effect (i.e., the completely standardized indirect effect). Thus, the latter two regressions were also needed. We ran all PROCESS analyses twice to obtain both 95% and 99% confidence intervals and we report the widest interval that was significant. Follow-up mediation analyses added in covariates (see Fig 1) by stipulating, in PROCESS, that the covariation was of both the mediator and the DV.

For all analyses there were no violations of normality, linearity, homoscedasticity, sequential dependence, independence, or multicollinearity. No univariate or multivariate outliers were identified.

### Hypotheses 1 to 3

The zero-order correlations among variables are presented in Table 1 alongside the means, standard deviations, and ranges, while the estimates of the multiple regressions are presented in Table 2. The three multiple regressions each had the same predictors, attachment anxiety and attachment avoidance, but different dependent variables, being social anxiety, suppression, and cognitive reappraisal respectively.

**Table 1:** Correlations, means, standard deviations, minimums and maximums of the measured variables (N = 253).

		Correlations									Mean	SD	Min	Max
		2	3	4	5	6	7	8	9	10				
1	Attachment anxiety	.531**	-.321**	.199**	.323**	174**	.280**	.313**	.396**	.226**	2.99	1.28	1.00	6.50
2	Attachment avoidance		-.153*	.478**	.248**	.123	.194**	.308**	.276**	.269**	3.02	1.29	1.00	6.28
3	Cognitive reappraisal			-.121	-.357**	-.248**	-.346**	-.316**	-.329**	-.262**	4.80	1.31	1.00	7.00
4	Suppression				.231**	.042	.125*	.309**	.288**	.309**	3.56	1.41	1.00	7.00
5	Social Anxiety					.872**	.902**	.661**	.754**	.782**	2.47	.72	1.00	4.43
6	Giving Criticism						.817**	.359**	.466**	.576**	22.33	6.96	7.00	35.00
7	Expressing an Opinion;							.488**	.653**	.594**	15.51	5.69	6.00	30.00
8	Giving a Compliment								.607**	.635**	6.56	2.75	4.00	17.00
9	Initiating Contact									.548**	11.01	4.11	5.00	25.00
10	Positive Self-Evaluation										10.24	3.78	4.00	20.00

* *p* < .05

** *p* < .01.

**Table 2:** Multiple regressions of attachment anxiety (Att Anx) and attachment avoidance (Att Avoi) predicting, in three separate regressions, social anxiety, suppression, and cognitive reappraisal (Cog Reapp) (N = 253).

Dependent variable	*R*	Predictors	*b*	*SE b*	*β*	*p*	*r*	*sr*
Social Anxiety	.335	Att Anx	.149	.039	.266	< .001	.323	.226
		Att Avoi	.059	.039	.106	.132	.248	.090
Suppression	.482	Att Anx	-.084	.072	-.076	.245	.199	-.065
		Att Avoi	.564	.071	.519	< .001	.478	.439
Cog Reapp	.322	Att Anx	-.340	.072	-.334	< .001	-.321	-.283
		Att Avoi	.024	.071	.024	.733	-.153	.020

*b* = unstandardized beta, *β* = standardized beta, *SE* = standard error, *r* = zero-order correlation, *sr* = semi-partial correlation. For clarity, the leading zeros are omitted for *b* and *β*.

Social anxiety was positively associated with attachment anxiety (*r* = .323, *r*^*2*^ = 10.4%, *p* < .001) and attachment avoidance (*r* = .248, *r*^*2*^ = 6.2%, *p* < .001) according to the zero-order correlations (see Table 1). The association between attachment anxiety and social anxiety was not significantly greater than the association between attachment avoidance and social anxiety *z* = 1.293, *p =* .196. H1 was, therefore, only partially supported. The multiple regressions showed that once the variance explained by attachment avoidance was removed, attachment anxiety still accounted for the variance in social anxiety (*sr*^*2*^ = 5.1%; the unique variance), but only half as much of it. By contrast, attachment avoidance only uniquely explained a small amount of the variance (*sr*^*2*^ = 0.8%) in social anxiety (i.e. with the variance accounted for by attachment anxiety was removed). This suggests that for attachment avoidance, at least, the variance it explains in social anxiety largely overlaps with the variance of attachment anxiety; the same did not hold for attachment anxiety, which still–uniquely–explained variance in social anxiety.

Suppression was found, according to the zero-order correlations at least, to be positively associated with both attachment anxiety (*r* = .199, *r*^*2*^ = 4.0%, *p =* .001) and attachment avoidance (*r* = .478, *r*^*2*^ = 22.8%, *p* < .001). The association between attachment avoidance and suppression was significantly greater than the association between attachment anxiety and suppression *z* = -5.017, *p* < .001. H2 was, therefore, supported. The multiple regressions highlighted that–uniquely–attachment anxiety explained little of the variance in suppression (*sr*^*2*^ = 0.4%), while attachment avoidance still, even uniquely, accounted for a substantial amount of the variance (*sr*^*2*^ = 19.3%; i.e. even with the variance explained by attachment anxiety removed). So when predicting suppression, the variance accounted for by attachment anxiety seems largely that of attachment avoidance; the opposite of what we observed with social anxiety as the dependent variable.

Cognitive reappraisal was found to be negatively associated with both attachment anxiety (*r* = -.321, *r*^*2*^ = 10.3%, *p* < .001) and attachment avoidance (*r* = -.153, *r*^*2*^ = 2.3%, *p* = .015) when using zero-order correlations. The association between attachment anxiety and cognitive reappraisal was significantly greater than the association between attachment avoidance and cognitive reappraisal *z* = -2.860, *p* = .004. H3 was, therefore, supported. The unique variance of the multiple regressions showed that while attachment anxiety still accounted for variance in cognitive reappraisal (*sr*^*2*^ = 8.0%), attachment anxiety accounted for virtually none (*sr*^*2*^ = 0.0%); so similar to what we observed for social anxiety.

### Hypothesis 4 and 5

For the first mediation with attachment anxiety as the IV, the correlation with social anxiety (i.e., the total effect) explained 10% of the variance. This first mediation had two indirect paths (i.e., two mediators). The first indirect path, via cognitive reappraisal, comprised of correlations between cognitive reappraisal and both attachment anxiety and social anxiety, explaining 10% and 13% of the variance, respectively. The second indirect path, via suppression, comprised of correlations between both suppression and attachment anxiety and social anxiety explaining 4% and 5% of the variance, respectively.

For the second mediation with attachment avoidance as the IV, the correlation with social anxiety (i.e., the total effect) explained 6% of the variance. This second mediation also had two indirect paths. The first indirect path, via cognitive reappraisal, comprised of correlations between cognitive reappraisal and both attachment avoidance and social anxiety explaining 2 and 13% of the variance, respectively. The second indirect path, via suppression, comprised of correlations between suppression and both attachment avoidance and social anxiety explaining 23 and 5% of the variance, respectively. The advantage of mediations over correlations are that they compute a single statistic for the indirect path allowing this path to be contrasted with the direct path.

The results of all mediations are reported in Table 3. The two aforementioned mediations (i.e., the initial mediations) had two mediators (i.e., cognitive reappraisal and suppression), and the same DV (i.e., social anxiety). However, in the first mediation the IV was attachment anxiety, whereas in the second mediation the IV was attachment avoidance, as illustrated in Fig 1. The first mediation showed the association between attachment anxiety and social anxiety was mediated by both mediators; that is, the two indirect paths were significant. However, these indirect paths, even in combination, only accounted for 37% of the association between the IV and DV ([*β*_indirect, reappraisal_ + *β*_indirect, reappraisal_]/ *β*_Total effect_, i.e., .119/.323). By contrast, the direct path accounted for 63% of the association between the IV and DV. This result is shown in the left-most bar of Fig 2, which plots the *β*s and exhibits the size of the indirect paths relative to the direct path. This emphasis on effect sizes is preferable to merely reporting whether the indirect paths are significant or not [77].

**Table 3:** The unstandardized (*b*) and standardized (*β*) beta weights of the four mediations.

					Total effect	Direct effect				Indirect effect
		DV	Covariate	Mediators (M)	*b*, *β*, *p*	*b*, *β*, *p*		IV-M *b*	M-DV *b*	*b (ci*, 95 or 99%), *β*
Attachment anxiety as the IV										
	Initial mediation (no covariate)	Soc Anx	None	Cog Reapp & Supp	.181, .323, < .001	.114, .204, .001	Cog Reapp Supp Total	-.327 .218	-.150 .080	.049 (.015 to .096, 99), .088 .018 (.002 to .041, 95), .031 .067 (.025 to .121, 99), .119
	Follow-up mediation (with covariate)	“	Att Avoi	“	.149, .266, < .001	.104, .185, .009	Cog Reapp Supp Total	-.340 -.084	-.151 .073	.051 (.013 to .105, 99), .092 -.006 (-.021 to .004, 95), -.011 .045 (< .001 to .107, 99), .081
Attachment avoidance as the IV										
	Initial mediation (no covariate)	“	None	“	.138, .248, < .001	.077, .138, .036	Cog Reapp Supp Total	-.155 .520	-.179 .064	.027 (.004 to .059, 95), .049 .034 (-.005 to .075, 95), .060 .061 (.016 to .110, 95), .109
	Follow-up mediation (with covariate)	“	Att Anx	“	.059, .106, .132	.022, .039. .600	Cog Reapp Supp Total	.024 .564	-.151 .073	-.004 (-.027 to .021, 95), -.007 .041 (-.001 to .087, 95), .074 .037 (-.011 to .087, 95), .067

The indirect effects were computed using bootstrapping of 1,000 resamples. Att Anx = Attachment anxiety, Att Avoi = Attachment avoidance, Soc Anx = Social anxiety, Cog Reapp = Cognitive reappraisal, Supp = Suppression. For clarity, the leading zeros are omitted for *b* and *β*.

The second of the initial mediations included attachment avoidance as the IV. This analysis showed that the association between attachment avoidance and social anxiety was, again, mediated. While the load carried by the two indirect paths was comparable, as illustrated by the *β*s in the third bar across in Fig 2, only one of the two mediators (i.e., cognitive reappraisal) was significant. Collectively, these indirect paths accounted for 44% of the association between the IV and DV, compared to 56% for the direct path.

Given that attachment avoidance and attachment anxiety are significantly correlated (*r* = .53), it is possible that the results of one of the initial mediations reported above is simply an artefact of this association. To rule out this possibility, we ran two follow-up mediations that assessed whether the above findings remained after controlling for the other attachment style. The first follow-up mediation (Fig 1A) consisted of attachment anxiety as the IV and attachment avoidance as the covariate. By contrast, the second follow-up mediation (Fig 1B) consisted of attachment avoidance as the IV and attachment anxiety as the covariate as shown in Fig 1 and Table 2. Compared to the results of the initial two mediations, the consequences of including the covariates in the follow-up mediations was, broadly speaking, that the total effects were diminished but, importantly, the ratios of the direct to indirect effects remained broadly similar. We illustrate the impact of the covariates in two ways. First, with the *β*s of Fig 2, which show that with the covariate added the reduction in the total effect is only slight for the mediation with attachment anxiety as the IV but is substantial for the mediation with attachment avoidance as the IV. Fundamentally, this is accounted for by a large reduction in the direct effect, with the covariate added, for the latter but not the former of the two mediation. Also, to a lesser extent, one of the mediators also becomes tiny and negative in each mediation (suppression and reappraisal respectively). So for the mediation with attachment anxiety as the IV the indirect effect and one direct effect remains significant, while for the mediation with attachment avoidance as the IV, no paths are significant. Second, by contrasting the percentages of variance explained, which is an effect size on a different scale to the *β*s the reported above. For attachment anxiety the total effect decreased from 10.4 to 5.1% of the variance with the covariate added. By contrast, for attachment avoidance the total effect decreased from 6.2 to 0.8%.

These follow-up mediations suggest that for attachment avoidance, the direct (and indirect) effect we initially observed were driven by the portion of attachment avoidance that was *correlated* with attachment anxiety. After removing this portion there were no direct (or indirect) effects resulting from the portion of attachment avoidance that was *independent* of attachment anxiety. By contrast, for attachment anxiety, the direct and mediation effects were due largely to variance specific to attachment anxiety and not to variance shared with it and attachment avoidance. The results, therefore, partially support H4 but not H5.

## Discussion

This study explored the relationship between insecure attachment dimensions (i.e., avoidance and anxiety), emotion dysregulation, and symptoms of social anxiety. In accordance with H1, attachment avoidance and anxiety were positively related to interpersonal discomfort (i.e., social anxiety). This finding is the first, to our knowledge, to report the associations between attachment anxiety and attachment avoidance dimensions and social anxiety symptoms and suggests that both attachment dimensions may be relevant to understanding social anxiety symptomology. This finding is consistent with the relatively small body of existing studies which have evaluated social anxiety in relation to categorical measures of attachment security, which have suggested that insecure attachment is associated with stronger symptom severity in social anxiety [10,35,36]. Additionally, the present study found that higher levels of attachment anxiety and avoidance were associated with higher levels of social anxiety symptoms across a range of interpersonal (i.e., social evaluative) situations. This finding is in line with previous research highlighting associations between insecure attachments and excessive emotional discomfort experienced within interpersonal situations [26,37,38].

The second hypothesis was supported; that is, attachment avoidance and anxiety were positively associated with the use of suppression. A stronger association was found between attachment avoidance and the use of suppression compared to attachment anxiety and the use of suppression. The differences in the strength of these relationships supports previous studies (e.g., [61]) showing attachment avoidance to be more strongly associated with the suppression of attachment related thoughts when compared to anxiously attached individuals.

In support of our third hypothesis, cognitive reappraisal was negatively associated with both attachment avoidance and anxiety. This finding is consistent with the attachment theory assumption that anxious and avoidant attachments are associated with deficits in the ability to generate effective emotion regulation strategies [21].

More notably, support was obtained for our fourth hypothesis, providing evidence for the mediating roles of both cognitive reappraisal in the relationship between attachment anxiety and interpersonal discomfort (i.e., social anxiety). This result suggests that individuals with high levels of attachment anxiety do not employ adaptive emotion regulation strategies (i.e., lower reappraisal), which perpetuates attachment-related distress, and that the relationship between attachment anxiety and social anxiety may operate through emotion regulation. Accordingly, it can be said that attachment anxiety is an important factor that affects emotion regulation and patterns of interpersonal behavior, which determine an individuals’ social anxiety levels. The finding that reappraisal partially mediated the relationship between anxious attachment and social anxiety supports previous research (e.g., [64,65]) suggesting that anxiously attached individuals exhibit deficits in their ability to modify negative thoughts when confronted with situations that are interpreted as socially threatening.

Contrary to our expectations, results did not support our second proposed hypothetical causal chain outlined in H5. Thus, no significant indirect effects of attachment avoidance on social anxiety through the mediating roles of both cognitive reappraisal and suppression emerged from the data. Although we initially found significant indirect effects for cognitive reappraisal, further analysis revealed that this effect was driven by the portion of attachment avoidance that was correlated with attachment anxiety. Thus, the mediation effect disappeared once these effects were partialled out. The contribution of attachment avoidance to social anxiety symptoms may reflect an unwillingness to acknowledge or experience negative emotions [27,28], which may have been reflected in responses on self-report measures used in the present study. Put simply, the present study’s results show that attachment avoidance could be a risk factor in the development of social anxiety. However, variables other than the emotion regulation strategies assessed in the present study may be likely to be involved in explaining this relationship.

Results of the present study confirm that attachment anxiety and avoidance have a significant impact on social anxiety symptoms. More importantly, individuals with high levels of attachment anxiety experience, in part, social anxiety symptoms through the use of suppression and deficits in their ability to employ reappraisal, whereas individuals high in attachment avoidance do not. Thus, the hyperactivating strategies (e.g., emotional reactivity / hypersensitivity), and negative model of self, associated with attachment anxiety may perpetuate the use of suppression and deficits in the use of reappraisal resulting in higher social anxiety symptoms. This finding is consistent with research indicating that individuals who experience heightened anxiety are more likely to have negative internal working models of themselves [78].

To our knowledge, this is the first study to specifically evaluate the mediating effects of reappraisal and suppression on the relationships between attachment and social anxiety symptomology. The present results extend previous research by showing that, not only is attachment anxiety and avoidance associated with different and distinct attachment strategies for regulating distress [24,28,59], but they also differentially influence an individual’s experience of social anxiety through these different strategies. Specifically, attachment anxiety’s contribution to social anxiety symptoms was partially mediated by higher suppression and lower reappraisal, whereas these mediating roles were not found in the relationship between attachment avoidance and social anxiety.

The present study’s findings could have some important implications for the psychological treatment of social anxiety disorder. Cognitive Behaviour Therapy (CBT) is the leading evidence-based treatment of social anxiety disorder. A primary goal of CBT for social anxiety is to identify and challenge maladaptive cognitions reflecting the fear of negative evaluation through cognitive and behavioural strategies, with particular emphasis on exposure to anxiety-provoking situations with the aim of recognising that feared outcomes do not occur [79]. Exposure techniques are delivered individually [80] or in a group format [81] and may involve graded exposure to situations, thoughts or sensations that trigger symptoms of distress. In this framework, socially anxious individuals learn to identify and challenge automatic thoughts, modify distorted beliefs about the social self and restructure biased threat-related appraisals [82]. Assessing the attachment orientation of individuals with social anxiety may provide clinical insight into how the distress regulation strategies of those with anxious and avoidant attachments influence the efficacy of behavioural techniques and how such techniques are optimally delivered. With this information, therapists may develop an understanding of the interpersonal situations and emotion regulation strategies most likely to elicit social anxiety symptoms consistent with one’s particular attachment difficulties and modify exposure components of CBT protocols to improve treatment outcomes. Furthermore, research suggests a strong therapeutic alliance is associated with improved client engagement during exposure sessions for social anxiety [83] and individuals’ attachment may influence the strength of the therapeutic alliance [84]. Future research should therefore explore whether assessing individual attachment orientation could assist therapists in building a strong therapeutic alliance and whether such an understanding could help to improve treatment outcomes.

While the efficacy of CBT for social anxiety disorder has been established in multiple randomised control trials (e.g., [9,79]), there is evidence to suggest that treatment effect sizes are attenuated when compared to other cognitive behavioural interventions for anxiety-related difficulties such as obsessive compulsive disorder or generalised anxiety disorder [85]. There is, therefore, room for improvement in treatment outcomes. Additionally, treatment trials have primarily examined changes in social anxiety symptoms and diagnostic status, thereby meaning that improvement in social behaviour remains under-addressed [86]. Assessing and understanding how different attachment-driven emotion regulation strategies impact on social anxiety symptoms may have the potential to provide clinical insight into the specific interpersonal behaviour patterns employed by socially anxious individuals (e.g., interpersonal dependency behaviours associated with anxious attachments as opposed to avoidance behaviours associated with avoidant attachments). With this information, targeted interventions which incorporate social skills training could, potentially, be tailored to improve interpersonal functioning by modifying dysfunctional emotions, thoughts and behaviours in the context of interpersonal situations.

There is increasing recognition that intimate relationship quality influences the onset and maintenance of many forms of psychopathology [87,88]. Research suggests individuals who exhibit high social anxiety symptoms report less satisfying intimate relationships [6], experience diminished closeness to partners during times of distress [89] experience lower levels of intimacy [90], and are more likely to assign blame and responsibility for conflicts in their intimate relationships on stable traits of their partners when compared to non-socially anxious individuals [91]. Such difficulties may be seen to result from, and/or be exacerbated by, the behaviours associated with each attachment dimension. Given that negative interaction patterns in intimate relationships can exacerbate individual psychological problems and hinder the effectiveness of psychological treatment [92] the results of the present study would suggest that considering relationship satisfaction and functioning in relation to the variables examined in this study would be of importance. Furthermore, evidence suggests that the involvement of significant others in the treatment of mental health disorders may improve mental health and relationship outcomes [87]. Consequently, future studies should also explore whether the inclusion of attachment-focused couples-based interventions (e,g., Emotion-Focused Couples Therapy, EFT; [93]) as a component of the treatment of social anxiety could improve patient anxiety and relational outcomes. For instance, an intervention such as EFT could help patients to understanding the distress regulation strategies consistent with one’s particular attachment difficulties (e.g., hyperactivating strategies associated with anxious attachments and deactivating strategies associated with avoidant attachment) and their associated use of suppression and cognitive reappraisal. This insight could assist in modifying negative working models and increasing security in the attachment bond, which in-turn may reduce symptoms of social anxiety for those who are in an intimate relationship. However, the precise relationship between the dimensions of attachment and social anxiety symptomology would benefit from further elucidation before these findings being utilised to inform clinical practice.

### Limitations

Results derived from the present study should be interpreted in consideration of several limitations. Our sample was mainly comprised of females (79.8%). Comparable levels of over-representation of female participants has been observed in a range of psychological studies employing online surveys which have utilised the same or similar recruitment methods to the present study (e.g., [33,94]). The fact that a similar gender skew has been observed across the range of psychological studies suggests that the over-representation of female participants from undergraduate and online community research pools may not be due to the subject matter of the study or the variables being investigated. Nevertheless, caution must be taken in generalizing these findings to other populations.

Although we did not obtain a clinical sample of individuals with social anxiety, we were able to obtain a range of participants who exhibited varying levels of social anxiety symptomology, including those with symptoms equivalent to samples of patients with social anxiety disorder. Thus, the generalizability of our findings must be examined in future research with clinical populations. Another limitation of this present study is its cross-sectional research design. Although there is theoretical value in analysing findings that were acquired from adults at a single point in time, the findings of the present study do not fully reflect the complexities of the attachment system over time. Similarly, as a cross-sectional design, the causality implied by the mediation model must be interpreted with caution and future validation of this mediation relationship should involve assessment of the variables at multiple time points (e.g., [95]). Future research should use longitudinal designs to enable a better understanding of the relationship between attachment dimensions, emotion regulation, and social anxiety symptomology. Finally, the present study relied entirely on self-report measures, which are subject to bias [96] and did not include any objective measures. Therefore, it is possible that the cognitive biases and affect-regulation strategies associated with insecure attachments, such as underreporting/minimisation of distress in individuals with high attachment avoidance (e.g., [97]) may have impacted upon our results.

## Conclusion

It can be concluded from the findings of the present study that there are significant relationships between both insecure attachment dimensions and social anxiety symptomology. The present study adds to the attachment literature by providing a more informed understanding of how variations in attachment systems are linked to the processing of attachment relevant information across a range of interpersonal domains. More importantly, results suggest that attachment anxiety and attachment avoidance impact upon indices of social anxiety symptomology. However, the use of reappraisal were found to partially mediate the relationship between attachment anxiety and social anxiety. Thus, it can be tentatively concluded that negative early attachment experiences may foster the development of attachment anxiety, which perpetuates the use of dysfunctional emotion regulation strategies, resulting in heightened social anxiety symptoms. Results emphasise the need for future research to investigate other variables that may contribute to the relationship between insecure attachment dimensions and social anxiety.

## Supporting information

S1 File Data file. (SAV)
